# The effect of spinal manipulative therapy on heart rate variability and pain in patients with chronic neck pain: a randomized controlled trial

**DOI:** 10.1186/s13063-019-3678-8

**Published:** 2019-10-12

**Authors:** Anders Galaasen Bakken, Iben Axén, Andreas Eklund, Søren O’Neill

**Affiliations:** 1 Intervention and Implementation Research for Worker Health, Institute of Environmental Medicine, Karolinska Institutet, Nobels väg 13, S-171 77 Stockholm, Sweden; 2 University of Southern Denmark, Campusvej 55, DK-5230 Odense, Denmark

**Keywords:** Pain, Heart rate variability, Conditioned pain modulation, Spinal manipulative therapy, Chiropractic, Neck pain

## Abstract

**Background:**

Recent experimental research has suggested that spinal manipulative therapy (SMT) may reduce pain through modulation of the ascending pain signals and/or the central pain-regulating mechanisms. People with persistent neck pain (NP) have also been found to have disturbances in autonomic nervous system (ANS) regulation. A common way to study the ANS is to measure heart rate variability (HRV). It is not known whether deviations in HRV are related to changes in pain perception or to the treatment response to SMT.

Commonly, an individual in pain will experience pain reduction when exposed to a second pain stimulus, a mechanism known as conditioned pain modulation (CPM). Patients with persistent pain have been found to have a reduced CPM reaction. It is not known whether this is predictive of treatment response to SMT.

The aim of the study is to examine the effects of SMT on HRV and pain. Further, a secondary aim is to test whether a CPM test can be used to predict treatment response in a population of patients with recurrent and persistent NP.

**Method/design:**

A multicentre randomized controlled clinical trial will be carried out in multidisciplinary primary care clinics. This setting is chosen to minimize bias resulting from patient preference for the treatment modality and provider.

The subjects are either self-referred or referred from other health care practitioners locally. The treatment modalities are two well-known interventions for NP; SMT and stretching exercises compared to stretching exercises alone.

HRV will be measured using a portable heart monitor. The subjective pain experience will be investigated by assessing pain intensity and the affective quality of pain. CPM will be measured with a standardized cold pressor test. Measurements will be performed three times during a 2-week treatment series.

**Discussion:**

The study will utilize normal clinical procedures, which should aid the transferability and external validity of the results. The study will provide knowledge regarding the underlying mechanisms of the effects of SMT. Furthermore, the study will examine whether a CPM test is predictive of treatment outcome in a population of patients with recurrent and persistent NP.

**Trial registration:**

ClinicalTrials.gov, NCT03576846. Registered on 3 July 2018.

**Electronic supplementary material:**

The online version of this article (10.1186/s13063-019-3678-8) contains supplementary material, which is available to authorized users.

## Background

Musculoskeletal pain is a global burden due to a high prevalence and substantial costs worldwide [1]. The most common conditions are low back pain (LBP) and neck pain (NP) [2]. Despite years of research, diagnosing LBP and NP is still difficult, and up to 90% of cases are termed “non-specific”. This often results in treatments that are based on untested theories (e.g. theories of spinal dysfunction, instability or muscle weakness) but also on the preference of the health care provider and patient. Ideally, the diagnosis should rest upon an understanding of the pain mechanism [3], but due to the “non-specific” diagnosis of LBP and NP, a therapist will often not be able to select the most appropriate treatment for the individual patient. This may be the explanation for the moderate treatment effect sizes for most available treatments, potentially wasting resources and failing to improve patients’ health [4].

Chiropractic care including spinal manipulative therapy (SMT) has been found to be a safe, effective and cost-effective non-invasive treatment for some types of spinal pain [5–7]. SMT has both local and regional pain-reducing effects [8], as well as central nervous system effects such as a general reduction of pain sensitivity [9].

SMT is thought to decrease pain by mechanically affecting muscular and joint function (i.e. normalizing muscle tone and improving joint mobility). However, recent experimental research has suggested that SMT may also be influencing the incoming/ascending pain signals (local nociceptive input affecting dorsal horn excitability or temporal summation) and/or the excitability of the central pain regulating mechanisms [9, 10]. A systematic review concluded that short-term sympathetic upregulation can be found with SMT, regardless of the spinal area being treated [11]. This raises the question of whether the pain-reducing effect of SMT is associated with a modulation of autonomic nervous system (ANS) activity.

Differences in ANS activity have been found between healthy controls and people with NP [12]. In healthy individuals, acute pain results in an increased sympathetic response and often an increase in pain threshold induced by descending inhibition. However, in patients with chronic pain, it appears that persistent sympathetic activation could lead to hyperalgesia due to a decrease in descending inhibition [12]. Further, in a recent study, chronic pain was reduced after treatment aimed at normalizing the ANS through biofeedback [12]. This indicates a bidirectional relationship; ANS not only reacts to pain, but pain is modulated by ANS activity. Furthermore, two studies [13, 14] (without control groups) have shown an association between positive treatment effects on pain and an increased heart rate variability (HRV). HRV is mainly considered a proxy for ANS regulation, as it will depend on the balance in the autonomic system.

Stretching is used both as a passive treatment intervention and as active home exercises for several conditions relating to the musculoskeletal system. The rationale behind stretching is to improve the range of motion and to reduce pain and perceived stiffness [15]. The effect of stretching exercises in combination with other treatment modalities has been found to be a decrease in pain and disability in patients with NP [4, 16].

A pure placebo trial is not indicated either when an evidence-based treatment options exists or when the patients taking part in the study are actively seeking care [17]. Due to this, all patients in this trial are given home stretching exercises to ensure that some care is provided. One previous study showed that SMT had a greater effect on pain in combination with home exercises [18]. Using this design, the result will show whether adding SMT to stretching will yield different HRV and pain responses.

As already mentioned, chiropractic treatment is considered non-invasive and safe [5–7]. Common benign and short-lasting reactions to SMT are mild to moderate increases in pain in the area of treatment often coupled with fatigue [19], considered “normal reactions”. It has been shown that a normal reaction to treatment is a predictor for a good outcome [20], but the mechanism behind this is not known and appears not to have been previously studied or described in the literature. It may be hypothesized that the mechanisms behind normal reactions following SMT may also be explained by ANS reactions.

The research in the area so far suffers from some limitations: small group sizes, possible patient bias (positive expectations) towards the therapist and treatment method, short follow-up time and lack of a reasonable comparator treatment. A randomized design with a standardized control treatment would distinguish treatment effects from contextual effects. In order to study the neurological effect of SMT and stretching exercises compared to stretching alone in patients with persistent and recurrent NP in a clinical setting, a sufficiently large sample and a randomized design should be used. In order to study long-term HRV responses, measurements over 2 weeks will be conducted.

Interestingly, when exposed to pain, a different noxious (painful) stimulus can be used as a conditioning (inhibitory/facilitatory) stimulus. The normal reaction is a reduction in pain perception known as “pain inhibits pain” or inhibitory conditioned pain modulation (CPM). In patients with chronic pain, a reduced CPM response may prevent the normal reaction to a painful stimulus from occurring, and patients will not experience the normal “pain inhibits pain” reaction [21].

In this study, a tool previously described in the literature [22] will be used to test the CPM response. The aim is to study whether patients with reduced CPM prior to starting treatment will respond differently to a series of chiropractic treatments than patients with functioning CPM; that is, to study whether a test for CPM and its results can be used as a predictor of treatment outcome. The cold pressor test (CPT) is known to be a safe testing method with minimal adverse reactions [23].

## Method/design

### Study aim

The study aim is to determine the effects of a treatment series consisting of stretching and SMT versus stretching alone on HRV and pain in a clinical setting in a population of patients with recurrent or persistent NP. A secondary aim is to test CPM as a predictor of treatment outcome in terms of pain.

### Setting

This multicentre randomized controlled clinical trial will be carried out in multidisciplinary primary care clinics where physiotherapists and chiropractors are consulted for musculoskeletal pain. These types of clinics are selected to minimize bias from patients having expectations towards a specific treatment modality.

A total of six clinics will take part in this study, and each clinic will include 20 subjects, resulting in 120 subjects in total (60 in each treatment arm).

Two research trained clinicians will conduct all of the measurements.

The treatments (both intervention and control) will be delivered by clinicians (licensed chiropractors) in the participating clinics.

### Eligibility criteria

Inclusion criteria: minimum 18 years old, able to read and understand Swedish, presence of recurrent (at least one previous episode) and persistent (duration more than 6 months) NP, and no chiropractic treatment during the previous 3 months. This interval was chosen as research has shown that similar treatments seem to have little effect beyond 3 months [24].

Exclusion criteria: conditions or medications that will affect the HRV measurements, such as cardiovascular disease, hypertension, diabetes, pregnancy, obesity (BMI > 30), currently using pain-reducing medication on a daily basis, steroids, β-blockers or antidepressants. All contraindications to SMT—that is, anything that could seriously aggravate the pain (e.g. inflammatory conditions) or be indicative of cerebrovascular injuries (previous drop attacks or a recent episode of a new headache or dizziness)—will exclude the patient from the study.

### Procedure

**Fig. 1:**
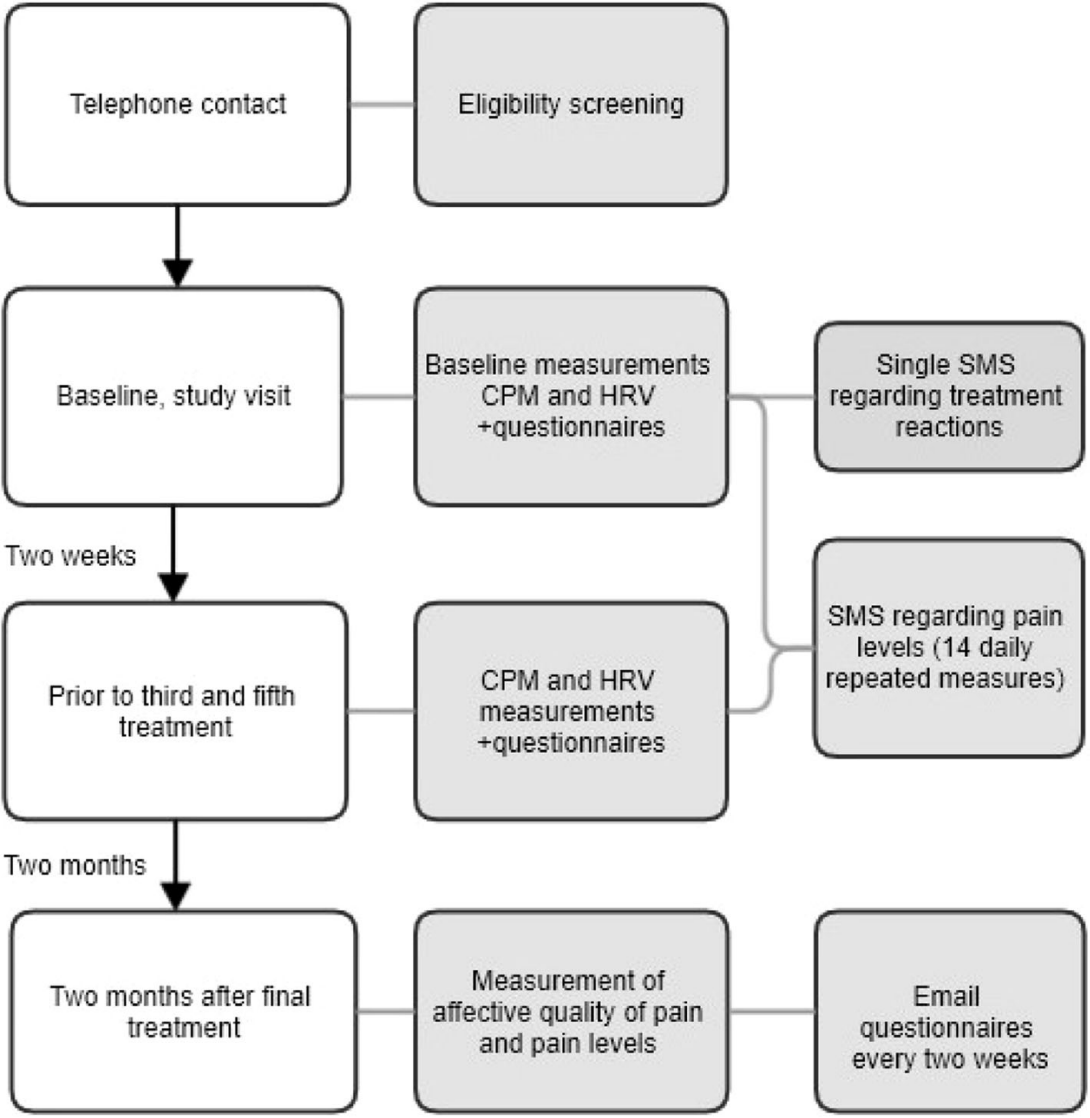
Flow chart of the study procedure. CPM conditioned pain modulation, HRV heart rate variability, SMS text messages

**Fig. 2:**
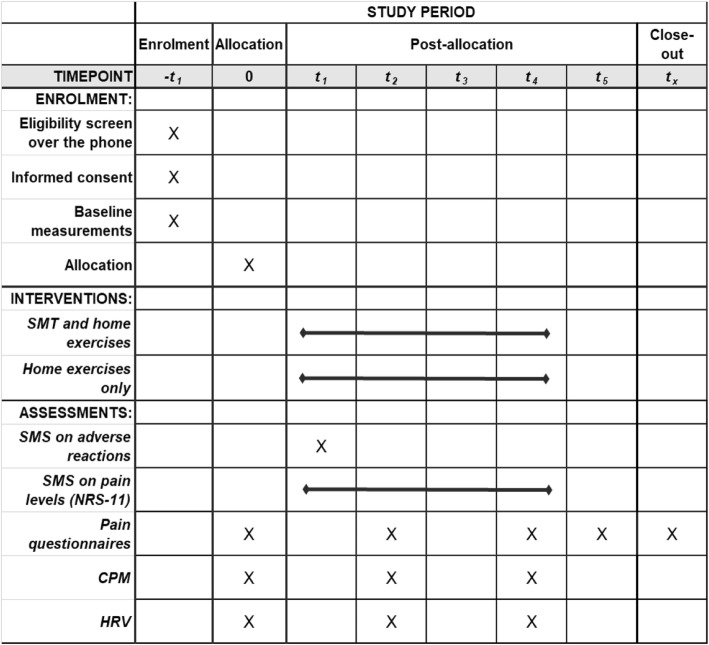
Spirit figure. CPM conditioned pain modulation, HRV heart rate variability, NRS-11 numerical rating scale (11 steps, 0–10), SMS text messages, SMT spinal manipulative therapy

The study procedure is shown in the flow chart and SPIRIT figure (Figs. 1 and 2).

#### Recruitment

Patients in this study are self-referred after hearing about the study from another health care provider or reading about the study in an advertisement or newsletter. A research assistant calls the patient and assesses their eligibility using a standardized form. The patients are informed about the aim of the study and the study procedures. If eligible, the patient is scheduled for all study visits during this call. Logistical details of this recruitment stage will be adapted to individual clinics as some clinics have newsletters and some use social media to inform their patients about clinic news. Different local newspapers are also used to recruit patients, as the individual clinics are located in and around the Stockholm area.

#### Study visit

On their first visit, patients will sign an informed consent form and have their baseline measurements taken. They will then be randomized to one of the treatment arms and treated accordingly. On the day of the study visit, the participants will refrain from caffeine, nicotine and alcohol, and from performing strenuous exercise.

A standardized protocol is followed on the day of the first study visit. After consenting to participate, the subjects will start by filling out a baseline questionnaire for demographic information. They will also answer questions concerning their NP (duration, episodes, intensity and frequency) as well as questions regarding pain levels and the affective quality of pain using the NRS-11 scale [25, 26], STarT Back [27, 28] and the short-form McGill Pain Questionnaire [29, 30]. Pain will be measured as average pain over the last 24 h. This information is collected on paper at the first visit, and transferred to a secure server at Karolinska Institutet (KI) by a research assistant. The follow-up questionnaires are digital, administered through Karolinska Institutet and managed by Survey & Report by Artologic (https://www.artologik.com/en/SurveyAndReport.aspx).

#### Measurements

The equipment used to measure HRV is called FirstBeat (https://www.firstbeat.com/en/). The monitor is applied by the research clinician, and the participants will rest quietly for 5 min with the equipment attached before baseline resting values are ascertained over a period of 5 min. After this, CPM will be tested with a structured CPM test [31]. This test includes mechanical pressure point intensity and a cold pressor test [22] (see Additional file 3 for a full description of the measurement procedure). Reported pain measurements during the CPM test are noted on a paper form and transferred to a secure KI server by a research assistant. When patients leave, the HRV equipment will still be attached to their chest, so that a measurement can be done the following night to record HRV in their deepest sleep [32]. Data collected from the FirstBeat monitors are downloaded to a secure computer administered by Karolinska Institutet.

After the measurements, study subjects are randomized into one of the two treatment arms. Their allocated clinician will conduct a standard anamnesis and examination procedure including neuro-orthopaedic tests to further assess the exclusion criteria. The treatment protocol is then initiated according to allocation. All subjects will be scheduled to a treatment series consisting of five visits over 2 weeks.

Data on normal treatment reactions (tiredness/fatigue and pain/tenderness) are collected the day after the first treatment using SMS (https://www.sms-track.com/) to ascertain the type and level of reactions to the interventions [33, 34]. The data are automatically stored in a secure cloud, accessible only by authorized researchers. For analysis, the data are transferred to a secure KI server.

Before the subjects’ third treatment, a second measurement of HRV and CPM will be conducted, and measurements of pain will be ascertained using the standardized protocol used at the initial visit.

Before the patients’ fifth treatment, or at least 2 days after the fourth visit, the final measurements will be conducted; this time, the HRV equipment will be taken off directly after the measurement. Again, the standard questionnaire measuring pain will be answered.

After the study period (four visits), the clinicians are free to select any other treatment modality for the patients. However, patients will be monitored with questionnaires every other week (via email) regarding their affective quality of pain and pain levels for 2 months after their final measurement at the clinic. The clinicians will also report what treatment modalities were used after the initial 2 weeks of the study.

Patients who do not complete the full treatment plan will be asked to complete all measurements in order to study attrition and to complete a drop-out analysis.

### Randomization procedure

Consecutively numbered opaque envelopes containing the group allocation are created off-site at the research centre by a statistician. A 1:1 allocation ratio in randomly permuted blocks of different sizes according to a randomization schedule is used. The envelopes are arranged in batches of 20 and distributed to the clinics at the start of each data collection period. SPSS version 20 is used to generate the randomization code. The envelopes are opened consecutively by the treating clinician.

### Blinding

The subjects will be unaware of what treatment the other group is receiving, as they will be told that the study is testing two different treatment modalities with similar clinical benefit to examine the effect over 2 weeks on physiological parameters and pain. Subjects in both treatment arms should feel that complying with their treatment plan during 2 weeks is a necessity for their improvement.

It will not be possible to blind the clinicians performing the treatments. The research clinicians who will collect the data in the experiment will be blinded to the treatment allocation. The statistical analysis will be performed with the treatment allocation blinded.

### Sample size

Log root-mean-squared successive differences in RR intervals (RMSSD) are the primary measurement of HRV. We will also explore other aspects of HRV according to Task Force Standards [35] to gain an overall impression of the subjects’ HRV. In a recent study that examined the reliability of HRV measures, the sample size was estimated as 20 subjects in each group to detect a mean change of 20% in RMSSD, and as 20–50 subjects in each group to detect a change of 10% [36]. A difference of 10–20% has been considered clinically important [36]. This value has also been used by other researchers investigating changes in HRV from manual treatment [37]. With a significance level of 5%, it was estimated that 60 subjects were needed in each treatment arm to reach a power of 80%. This is also in line with the general recommendations to detect a medium effect size [38]. A high number of drop outs is not expected in this study as it is conducted using an effective practice-based research network with established and tested routines developed to minimize the burden on participating patients.

### Treatment arms

SMT in this study is defined both as a high-velocity, low-amplitude (HVLA) thrust applied to the target joint and also as spinal mobilization (MOB) where the application of manual force to the spinal joints is within the passive range of joint motion and does not involve a thrust [39]. The type of techniques applied will be decided upon and described by the participating clinicians (chiropractors), and both HVLA and MOB will be considered manual treatments as they have been found to have similar effects on several pain parameters in a recent multicentre study [40]. This also provides the possibility for the chiropractor to adapt the force applied to the individual patient, which is normally done in the clinical encounter.

As the participating chiropractors have similar educational backgrounds and have received the same instructions concerning their limited choice of treatment techniques, we expect that they will have a similar approach to SMT. Data on the specific interventions will be collected.

The common modality used in both treatment arms is a programme of home stretching exercises. Both groups will receive verbal and written instructions describing the home stretching exercises that have been recommended as a low-cost, first-instance intervention for NP (Additional file 1) [16]. Patients will be instructed to keep an exercise diary to monitor their exercise frequency (Additional file 1) [16].

The testing of HRV and CPM will be conducted by two research clinicians only, to ensure consistency throughout the study. The two research clinicians will meet several times in advance of the study to test and calibrate the examination procedures and their communication with the study subjects.

### Outcome measures

#### Experimental measures

The primary outcome is log root-mean-squared successive differences in RR intervals (RMSSD). The variation in the beat-to-beat heart rate is an indicator of parasympathetic and sympathetic modulation of the heart rhythm. Deviations in HRV have been found in patients with both acute and chronic pain. Patients with various chronic pain conditions show reduced parasympathetic activity at rest, the proposed mechanism behind central sensitization [41]. Thus, vagus activity, assessed through HRV, is suggested to correlate with pain severity and could possibly be used as a proxy for treatment efficacy in patients with chronic pain [41]. There are some studies showing that SMT influences HRV, but the quality is questioned [42].

In this study, conditioned pain modulation (CPM) consists of the evaluation of a painful test stimulus followed by a second evaluation after the painful conditioning stimulus has been withdrawn (sequential stimuli) [21]. CMP is a well-known concept in modern medicine, particularly when it comes to prediction of post-operative pain [43]. It has been suggested that a dysfunctional CPM response can be a pathogenic factor in the development of chronic pain, but also that a dysfunctional CPM response can be the result of chronic pain, hence a possible bi-directional relationship [44].

There is a growing body of evidence suggesting that CPM may be an important biomarker of chronic pain and a predictor of treatment response [21]. One may suggest that in patients with chronic pain and a dysfunctional CPM response, treatments with approaches that address the central nervous system mechanisms (e.g. pharmacological and cognitive) could be the first choice of treatment. Patients with chronic pain that demonstrate a dysfunctional CPM might also be particularly sensitive to interventions that help to reduce the specific local nociceptive input (e.g. physical medicine and manual treatment). However, standardization of CPM testing is lacking [31].

Our study will use a structured CPM testing protocol with a standardized clamp pressing on the thumb nail for 10 s as the test stimulus, and cold water (0–2 °C) as the conditioning stimulus, previously tested and validated by O’Neill and O’Neill [22]. An NRS-11 will record pain associated with both stimuli. This allow us to examine whether the CPM responses are predictive of treatment outcomes after SMT and stretching exercises.

#### Patient-reported outcome measures

Secondary measurements such as disability and health-related quality of life will be collected. The subjective pain experience will also be evaluated, as this is important when considering chronic pain [45]. The outcomes will be collected using the following standard instruments:

- The neck disability index is an instrument designed to measure disability, and has been shown to be reliable and valid in Swedish [46].
- Pain intensity is measured with a validated NRS-11 where the subjects grade their perceived pain level using the anchors “no pain” and “worst possible pain” [25, 26].
- Measures of self-rated health are assessed by the EQ-5D, a translated (Swedish) and validated questionnaire with five domains and three answer options in each domain [47, 48].
- To assess the affective quality of pain, the Swedish version of the short-form McGill Pain Questionnaire-2 will be used. This is a validated questionnaire [29, 30] used in clinical trials designed to measure the subjective pain experience.

These questionnaires will be given at baseline, and again at the second and third measurements. In addition, they will be administered every other week during the following 2 months after the study period has ended. Pain intensity (NRS-11) [25, 26] will be collected daily during the 2-week study period using text messages (SMS) and every other week during the following 2 months using emailed questionnaires.

The most common side effects following SMT are local tenderness and tiredness of a short duration [19]. In this study, the reactions to both treatment arms will be monitored using SMS sent to the participants 1 day after the first treatment.

### Time line

The data collection commenced in January 2019 and is expected to be finished by February 2020. 

### Analysis

Intention-to-treat analysis will be applied.

Univariate multiple regression analysis (one outcome) and/or multivariate multiple regression analysis (more than one outcome) will be used to analyse the primary and secondary outcomes of the trial.

If appropriate, age and sex will be included as covariates and controlled for in the model.

To investigate whether CPM is a predictor of treatment outcome we will look at the statistical interaction between CPM and SMT with regards to their effects on the primary outcome.

### Ethical aspects

SMT is applied clinically in musculoskeletal health care and has been examined in a variety of research studies. Serious complications are very rare [49, 50]. The present study examines two commonly used treatment protocols in a clinical environment, which means that the subjects will not be subjected to a treatment that they would not normally receive when consulting for care. All test methods are well-established procedures commonly used in research practice. If a subject experiences an unexpected reaction to the treatment or testing procedure, the subject will be taken out removed from the study by the research assistant and will undertake an individual treatment plan.

The clinicians (chiropractors) who perform the treatment all have an academic degree, are licensed by the Swedish National Board of Health and Welfare (the national Patient Safety Act applies) and have personal liability insurance through their professional federation (https://www.lkr.se/) (Nordic Insurances). Thus, the subjects are insured in case of adverse events from treatment.

When screening for eligibility, both written and verbal information about the purpose of the study, treatments, measurements and SMS procedures will be provided. At the study visit, the patient will have an opportunity to ask the research clinician relevant questions, but will also be provided with a telephone number where a part of the research group not involved in the data collection can answer any questions they may have. All study subjects will sign informed consent forms before entering the trial.

Upon registration in the study, each subject will receive a subject identification number (ID), replacing their personal identity number and name, to which all measurement data and patient reported data will be linked. The key that matches the subject ID with their personal identity number and name will be securely stored in a locked fireproof cabinet at Karolinska Institutet in accordance with the National Board of Health and Welfare’s requirements for storage of journal documents.

During the data collection, data are recorded and processed by the research clinicians, and all entries in the databases are recorded using the subject ID only. During the analyses, data will be completely anonymized and only the involved researchers will have access to the data, which will be stored electronically at Karolinska Institutet in accordance with local rules and European GDPR regulations.

All reporting will be done at the group level without the possibility of identifying any individual study subjects. The results of the study will be published in open access journals, and will be communicated through several professional channels nationally and internationally.

Central ethical approval has been confirmed by the Regional Ethical Review Board in Stockholm (reference approval no. 2018/2137-31) and has approved participation of all individual centres in the trial.

## Discussion

The pain-reducing effects of SMT on certain spinal pain conditions are well established, as are the normal reactions to such treatment [5–7]. However, the mechanisms behind these effects are not well understood, although it is hypothesized that the pain-reducing effects could be mediated through the ANS [12]. Therefore, the study of HRV responses to SMT as part of a short treatment plan and its relation to pain sensitivity and normal reactions to treatment will advance knowledge regarding the mechanisms involved in the specific effects of SMT.

The development of CPM as a clinical prediction tool could potentially inform clinicians on what to expect in terms of treatment response concerning stretching and manual treatment. The knowledge gathered will inform future clinical studies regarding useful outcome measures, minimally clinically relevant change values and necessary sample sizes in this type of research.

There are some challenges to consider with the current design. The data collection demands effective recruitment to ensure a sufficient number of subjects to adequately power the trial. As mentioned, the study will utilize an existing practice-based research network, where chiropractors have participated and successfully recruited patients in previous studies, and thus we believe that it is feasible to include the required number of subjects.

Based on previous experience with multi-centre clinical trials, procedures are in place to minimize the burden on the subjects in the study. However, attrition is to be expected to some degree. Reminder functions have been included in the email and text-message measurement protocols, some of which are automatic, but some will require monitoring and individual follow up. As subjects deal with only one research clinician and one treating clinician, we believe that the personal contact will be optimal, thus reducing attrition.

The testing methods require a highly structured testing protocol as the testing equipment is highly sensitive. However, we believe that this is achievable considering the experience of the research group members.

A pilot study was conducted prior to commencing the full-scale study. This resulted in changes to the recruitment strategy with regards to the use of newspapers and advertising. The responsibility of booking eligible patients was transferred to a research assistant from the local receptionist.

## Trial status

Patient recruitment began in January 2019, and is expected to be completed by February 2020. 

## Additional files

Additional file 1: Stretching exercises, daily for 14 days. (DOCX 341 kb)

Additional file 2: SPIRIT Checklist. (DOC 123 kb)

Additional file 3: Protocol for measurements procedures (at all measurements). (DOCX 13 kb)

## Data Availability

All collected data will be stored for at least 10 years and can only be identified by code number. Only researchers who are directly involved in the project will have access to the material. Anonymized information may be shared with other researchers upon request, pending ethical approval.

## Abbreviations

ANS: Autonomic nervous system
CMP: Conditioned pain modulation
HRV: Heart rate variability
HVLA: High velocity, low amplitude
MOB: Spinal mobilization
NP: Neck pain
NRS-11: Numerical rating scale (11 steps, 0–10)
SMT: Spinal manipulative therapy
