# Supplementary material for: The effect of spinal manipulative therapy on heart rate variability and pain in patients with chronic neck pain: a randomized controlled trial
Source: Trials. 2019 Oct 12;20:590. doi: 10.1186/s13063-019-3678-8 (PMC6790043; doi:10.1186/s13063-019-3678-8)
Supplement: Supplementary file 1 — Stretching exercises, daily for 14 days. (DOCX 341 kb) [file 13063_2019_3678_MOESM1_ESM.docx]

# **Stretching exercises, daily for 14 days**

*The exercises must be done every day for 14 days. It will take approximately 10 minutes.*

*Please follow the description provided.*

*Use the exercise diary to make sure you remember to do your exercises*

**Sustain each stretch for 30 minutes and repeat 3 times**.

1. Bend your head to your left. Place your left arm on top of your head and feel the stretch.
2. Bend your head to your right. Place your right arm on top of your head and feel the stretch.


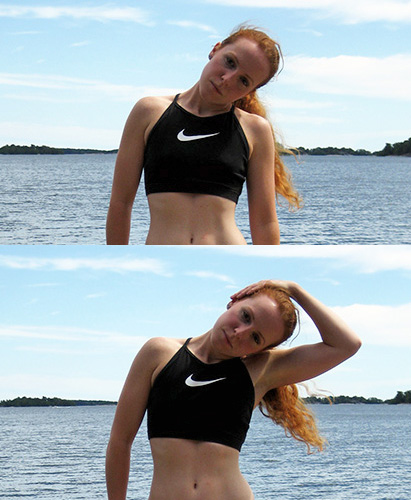


1. Bend and rotate your head to the left. Place your left arm on top of your head and feel the stretch.
2. Bend and rotate your head to the right. Place your right arm on top of your head and feel the stretch.


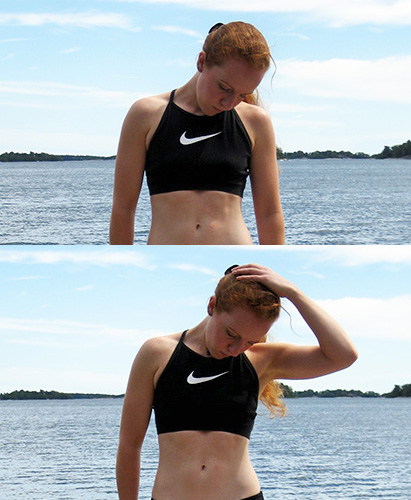


5: Bend your head forward. Place an arm on your head and feel the stretch.
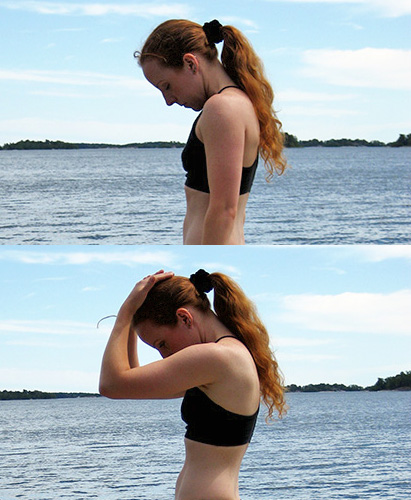


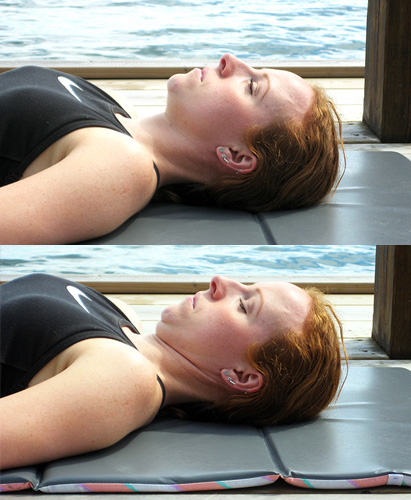
6: Finish off by sitting with your back straight, or lying flat on your back. Bring your chin back and hold for 3-5 seconds. Repeat 5 times.

Good luck!

## Date for your first consultation with the chiropractor:_______________

CODE: _____________

Circle your answer every day

Day 1: Stretching performed following the instructions Yes No

Day 2: Stretching performed following the instructions Yes No

Day 3: Stretching performed following the instructions Yes No

Day 4: Stretching performed following the instructions Yes No

Day 5: Stretching performed following the instructions Yes No

Day 6: Stretching performed following the instructions Yes No

Day 7: Stretching performed following the instructions Yes No

Day 8: Stretching performed following the instructions Yes No

Day 9: Stretching performed following the instructions Yes No

Day 10: Stretching performed following the instructions Yes No

Day 11: Stretching performed following the instructions Yes No

Day 12: Stretching performed following the instructions Yes No

Day 13: Stretching performed following the instructions Yes No

Day 14: Stretching performed following the instructions Yes No

Comments: _________________________________________________________

______________________________________________________________________________
