# Supplementary material for: The effect of spinal manipulative therapy on heart rate variability and pain in patients with chronic neck pain: a randomized controlled trial
Source: Trials. 2019 Oct 12;20:590. doi: 10.1186/s13063-019-3678-8 (PMC6790043; doi:10.1186/s13063-019-3678-8)
Supplement: Supplementary file 3 — Protocol for measurements procedures (at all measurements). (DOCX 13 kb) [file 13063_2019_3678_MOESM3_ESM.docx]

# Protocol for measurements procedures (at all measurements)

Prior to the first measurement, the patient is informed about the procedures.

1. The FirstBeat device is attached on the patient chest.
2. The patient is asked to sit on a chair facing the wall, with hearing protection to prevent any disturbing noise. They are asked to keep their eyes closed and breathe with normal pace for 10 minutes.
3. The first 5 minutes are used as relaxation to assure the same measuring conditions each time. After this, the start time of the measurement is registered by the research assistant, and the patient sits for another 5 minutes for the measurements to be taken.
4. After finishing the HRV measurement, the patient undergoes the CPM test. This is done by applying a clamp on the thumb nail for 10 seconds. The pain intensity is reported on a NRS-11 scale. The opposite hand is then placed in a bucket of cold water (0-2°C) for two minutes before a second test with clamp on the thumbnail, and pain intensity is reported as before on a NRS-11 scale.
5. The subject then meets their allocated chiropractor. (After the first measurement, the randomization envelope Is opened by the treating chiropractor.) The FirstBeat device stays attached to the patient, to be removed the following morning. This is done in order to get a measurement the night following the treatment. This is not done, however, for the final measurement where the device is detached straight after the measurement.
